# Investigation of the Effects of Selenium Against 4-Nonylphenol-induced Toxicity in Rat Testis

**DOI:** 10.1007/s12011-025-04539-8

**Published:** 2025-02-05

**Authors:** Gülsüm Yılmaz, Ülker Eren, Özay Güleş, Murat Boyacıoğlu

**Affiliations:** 1 Department of Histology and Embryology, Institute of Health Sciences, Aydın Adnan Menderes University, Aydın, Turkey; 2 Department of Histology and Embryology, Faculty of Veterinary Medicine, Aydın Adnan Menderes University, Işıklı, Aydın, 09012 Turkey; 3 Department of Pharmacology and Toxicology, Faculty of Veterinary Medicine, Aydın Adnan Menderes University, Aydın, Turkey

**Keywords:** Sodium selenite, 4-Nonylphenol, Caspase 3, Spermatozoa, Rat, Testis

## Abstract

The study aims to investigate whether selenium (Se) has a protective role against testicular toxicity induced by 4-nonylphenol (4-NP) in rats and reduces oxidative damage. For this purpose, 30 adult male Sprague–Dawley rats (250–300 g/90 days old) were divided into five equal groups: control, sham control, Se, 4-NP, and 4-NP + Se. The trial lasted 48 days, with 4-NP administered at 125 mg/kg/day and Se at 0.5 mg/kg/day. The general microscopic examination of the testicular tissue involved measuring the diameters of seminiferous tubules, epithelial heights, and the density of stage XIV tubules in sections stained with the triple staining method. Caspase 3 and CX43 expressions were observed immunohistochemically, and the numbers of live-dead and normal-abnormal spermatozoa were recorded. The levels of malondialdehyde (MDA) and superoxide dismutase (SOD) were determined in blood serum and testicular tissue. At the end of the study, testicular toxicity due to 4-NP was demonstrated cytologically, histologically, histometrically, biochemically, and immunohistochemically. Se showed a positive effect against this toxicity, as evidenced by higher stage XIV tubule density in the 4-NP + Se group, lower caspase 3 levels compared to the 4-NP group, decreased MDA levels, increased SOD levels in serum and testicular tissue, and a higher count of live and normal spermatozoa. When used alone, Se may cause metabolic adverse effects, such as decreased live weight gain, reduced tubule diameter and epithelial height, and increased caspase 3 expression, depending on the dose and duration of use.

## Introduction

Surfactants reduce the surface tension of water and establish a bridge between two chemicals that would not come together [1]. Therefore, surfactants form a group of various chemicals designed with cleaning and solubilizing properties [2]. Surfactants consist of two parts: a polar group, which is soluble in water, and a nonpolar hydrocarbon group, which is insoluble [2, 3]. Surfactants are classified as cationic, nonionic, or anionic based on the hydrophobic part of the molecules [3].

Nonionic surfactants, such as alkylphenol ethoxylates (APEs), are the most commonly used. Alkylphenols comprise two main compounds: nonylphenol ethoxylates (NPEOs) (80%) and octylphenol ethoxylates (OPEOs) (20%) [4]. In particular, NPEOs are used in household, industrial, or institutional detergents and emulsifiers, as well as in processes such as leather and textile treatment, latex paint, plastic, and paper production and the manufacturing of cleaning detergents. Some examples are also used in pesticide formulations [5]; therefore, industrial and domestic wastewater release NPEOs into the environment. Once released into the environment, they can transform into metabolic intermediates, including nonylphenol (NP), nonylphenol monoethoxylate, nonylphenol diethoxylate, and compounds formed through microbial transformation [6].

4-Nonylphenol (4-NP) is formed through the biological degradation of NPE. It is noteworthy for its ability to mimic the effects of female sex hormones and its disruptive impact [7]. Due to the benzene ring in its structure, 4-NP is resistant to biological breakdown (biodegradation) and prone to bioaccumulation [8]. The Environment Agency in the UK reported a biodegradation half-life of 150 days for 4-NP in water [9]. Shang et al. [10] reported a half-life of 60 years for NP in sediment.

The testis is a critical target organ for male reproductive toxicants, and exposure to such substances can lead to significant reproductive damage. 4-NP exerts its damaging effects through oxidative stress caused by excessive production of reactive oxygen species (ROS) [11, 12]. Treatment with 4-NP reduces body and testis weight [11] and induces histopathological changes in the testes, such as decreased diameter and epithelial height of seminiferous tubules [11, 13]. Excessive production of ROS can impair the normal functions of sperm, such as the acrosomal reaction, capacitation, and hyperactivation [12, 14]. These compounds have been shown to interfere with hormonal signaling pathways, particularly those involving androgen receptors. Lee et al. [15] reported that NP affects multiple steps in the activation and function of androgen receptors, inhibiting the binding of androgens, altering the nuclear localization of these receptors, and interfering with their interaction with coregulators, thereby impeding the transactivation process. Additionally, Liu et al. [16] suggested that the molecular mechanism of reproductive toxicity induced by NPs in Sertoli cells may be mediated through cell receptors and/or signal transduction pathways, with the effects varying depending on the isomeric side chain of NP compounds.

Selenium (Se) is a trace element found in the periodic table in Group VI A, existing in the air and dissolved in water, as well as in solid form in soil and rocks. It shares significant chemical similarities with sulfur, which also belongs to the same group. As it occurs in nature, selenium transfers from these sources to plants, fungi, bacteria, and humans, eventually returning to nature, thus completing a cycle [17].

Se is crucial for normal spermatogenesis in mammals, and its essential role is primarily mediated by two selenoproteins: phospholipid hydroperoxide glutathione peroxidase (PHGPx/GPx4) and selenoprotein P. PHGPx/GPx4 is the major selenoprotein expressed by germ cells in the testis, playing multiple roles and acting as the key link between selenium, sperm quality, and male fertility [18]. These selenoproteins are involved in redox-active regions, where selenium plays a critical role. Among them, glutathione peroxidase (GPx) is a key antioxidant enzyme responsible for reducing hydrogen peroxide to water within the cell. GPx contains one selenium atom in the form of selenocysteine in its subunit, making selenium a vital biological element [19]. Moreover, studies suggest that selenium, in interaction with vitamin E, protects cell membranes from oxidative damage caused by peroxides resulting from lipid metabolism [20]. Selenium supplementation treatment has been shown to reduce caspase-3 expression in germ cells increased due to electromagnetic field-induced testicular damage, and to increase the diameter and height of seminiferous tubules in damaged testis [21]. Furthermore, selenium has been found to decrease histopathological changes in testicular toxicity induced by monosodium glutamate [22] and bisphenol A [23]. Selenium supplementation has been shown to effectively prevent oxidative stress, inflammation, apoptosis, autophagy, and DNA damage in testicular toxicity induced by acrylamide in rats [24].

4-NP is an environmentally toxic substance with endocrine-disrupting effects that individuals of all ages may be exposed to. The present study aims to investigate whether selenium, a known antioxidant, plays a protective role in experimental testicular toxicity induced by 4-NP in rats and whether it aids in reducing oxidative damage resulting from toxicity. The literature review did not reveal any previous research on the effects of selenium in rats with testicular toxicity induced by NP.

## Materials and Methods

### Animals and Experimental Design

In the study, 30 adult male Sprague–Dawley rats (250–300 g/90 days old) were utilized as the study material. Throughout the research period, the rats were maintained under conventional conditions with a 12-h light/12-h dark cycle, providing ad libitum access to water and food. The environmental conditions were maintained at a temperature of 24 ± 1 °C and humidity at 50–55% throughout the study. All procedures were conducted following ethical guidelines (Ethical Committee Approval Decision no.: 64583101/2016/107).

The material was divided into five groups, with six rats used for each group. Rats were orally gavaged with 125 mg/kg/day dose of 4-nonylphenol (4-NP) (Acros-416240010) [25], dissolved in 0.5 ml corn oil [26]. Selenium (sodium selenite, Na_2_SeO_3_) (Sigma-214485) was administered at a dose of 0.5 mg/kg/day, dissolved in 0.5 ml physiological saline [26], also via oral gavage. For the 4-NP + Se group, Se was administered 1 h before 4-NP application. The experiment lasted for 48 days. No treatment was applied to the control group. The sham group received 0.5 ml corn oil and 0.5 ml physiological saline as a solvent throughout the experiment. To equalize the solvent effect, the 4-NP group, which used corn oil as a solvent, received physiological saline (the solvent for the Se group) in the same amounts, and vice versa for the Se group. All treatments were conducted in the mornings throughout the experimental period.

### Collection of Samples

At the beginning and throughout the trial, the body weights of both the control and experimental groups were recorded every 3–4 days.

After the completion of the treatment period, rats were fasted overnight (between 21:00 and 09:00, for about 12 h), and their live weights were determined the following morning. Blood samples were collected from rats anesthetized with xylazine-ketamine 24 h after the applications were concluded. Subsequently, testes were extracted from rats euthanized using cervical dislocation, following ethical guidelines. The weights of both testes were recorded separately. Tissue samples to be prepared from the right testes were fixed in Bouin’s solution for 24 h. After completing the fixation period in Bouin’s solution, tissue samples were sequentially placed in 50% alcohol for 48 h and 70% alcohol for 12 h. They were then transferred to 80% alcohol, followed by routine tissue processing and embedding in paraffin blocks. The left testes, designated for MDA and SOD analyses, were wrapped in aluminum foil and stored in a freezer at − 80 °C until the experimental stage.

In rats, the cauda epididymides were separated using a scalpel. The cauda epididymides were cut 5–6 times with scissors and incubated in 200-µl PBS drops at 35.5 °C for 30 min. This allowed for the diffusion of sperm into the PBS, and at the end of the incubation period, tissue fragments from the cauda epididymis were removed from the medium. This process resulted in obtaining a sperm suspension.

### Histological Method

Serial sections, 6 µm thick, were obtained from the prepared paraffin blocks at 200-µm intervals. Crossman’s triple staining method [27] was applied to six serial sections from each animal. The serial sections were first deparaffinized and rehydrated. The sections were then stained with hematoxylin for 10 min for nuclear staining, followed by a 10-min wash in running tap water. Next, they were stained with a mixture of acid fuchsin and orange G for cytoplasmic staining. The sections were transferred to 1% phosphotungstic acid in distilled water for 15 min until the connective tissue was decolorized. Finally, sections were stained with aniline blue for 1 min, followed by a 3-min immersion in 1% acetic acid to remove excess aniline blue. The sections were then dehydrated through three changes of absolute ethanol, cleared with three changes of xylene, and mounted with Entellan. The sections were subjected to general histological examination and histometric analysis, and nonspecific seminiferous tubular changes in the experimental groups were also evaluated [28].

### Determination of Histometric Changes

In the experimental groups, the diameter and epithelium height of stage VII-VIII tubules were determined. For this purpose, measurements were taken from six serial sections for each animal. In each section, 10 tubules, either round or nearly round in shape, were randomly selected. The diameter was measured four times for each tubule, and the height of the seminiferous epithelium was determined with eight measurements for each tubule.

To determine the density of spermatogenesis, the number of tubules belonging to stage XIV was identified in six sections for each animal, with a total of 30 tubules in each section. Measurements were conducted interactively using the Olympus BX43F research microscope and the Olympus cellSens Entry image analysis program.

### Immunohistochemical Method

The apoptosis rate and localization of Connexin 43 (a protein involved in gap junction communication) were determined using anti-caspase 3 (Sigma C8487) and anti-connexin 43 (Bioss bs-0651R) antibodies. For this purpose, the Strept ABC method was used [29]. Six sections from each animal were analyzed per antibody. Sections were deparaffinized and rehydrated, and antigen retrieval was performed in 0.01 M pH 6 sodium citrate buffer, heated at 100 °C for 5 min, and repeated three times. Endogenous peroxidase activity was blocked using 3% H_2_O_2_ for 15 min. Nonspecific background staining was blocked using a serum-blocking solution (Ultra V Block, Thermo Fisher Scientific) for 5 min.

The sections were incubated overnight at 4 °C with either anti-caspase 3 (1:200 dilution, Sigma C8487) or anti-connexin 43 (1:200 dilution, Bioss bs-0651R) antibodies. Following this, sections were incubated with biotinylated goat anti-polyvalent and then streptavidin-peroxidase for 5 min each. Afterward, all slides were treated with DAB (Sigma, D5637) for 2 min.

Sections were observed under an Olympus BX43F light microscope. For each animal, six serial sections were assessed, and 30 tubules were examined per section to determine the percentage of tubules with conspicuous caspase 3 positivity. CX43 positivity was evaluated subjectively, with photographs taken from the most intense areas.

### Biochemical Method

#### Tissue Homogenization

The left testes were cleaned by removing adherent connective tissue, and samples were diluted with a 10% solution of 150 mM phosphate buffer (pH 7.4) in an ice bath. Tissues were homogenized for 1 min at 2000 rpm using a blender (IKA-Werke GmbH and Co. KG, Staufen, Germany). The homogenate samples were then centrifuged at 7000 × g for 10 min at 4 °C (Hettich Zentrifugen, Mikro 200 R, Tuttlen, Germany), supernatants were separated, and testicular MDA and SOD levels were measured.

#### Total Protein Analyses

Total protein was employed in the calculation of MDA levels and SOD activity in the supernatants and serum. This analysis was performed using a commercially available kit (ArchemDiagnostik Ind. Ltd., Turkey) according to the Biuret method. Total protein was measured in a spectrophotometer (Shimadzu UV-1601, Kyoto, Japan) at 546 nm and expressed milligrams per milliliter.

#### Malondialdehyde (MDA) Measurement in Supernatants and Serum

Tissue lipid peroxidation levels were assessed according to the method described by Ohkawa et al. [30]. MDA analysis in serum was performed according to the method by Yoshioka et al. [31]. The analysis of supernatants and serum samples was performed at 535 nm, and results were expressed as nanomoles per milligram of protein. The MDA assay measures the concentration of malondialdehyde, a reactive aldehyde resulting from lipid peroxidation due to free radicals affecting cell membranes. The method relies on the spectrophotometric measurement of the color produced by the reaction between MDA and thiobarbituric acid. The concentration of MDA was calculated by the absorbance complex (absorbance coefficient *ε* = 1.56 × 105/M/cm).

#### Superoxide Dismutase (SOD) Measurement in Supernatants and Serum

Testicular tissues and serum samples of SOD activity were measured at 560 nm using a spectrophotometer and expressed as “units” per milligram of protein, following the method outlined by Sun et al. [32]. SOD enzymes catalyze the conversion of superoxide radicals to hydrogen peroxide. SOD estimation was based on the generation of superoxide radicals produced by xanthine on xanthine oxidase, which reacts with 2-(4-iodophenyl)−3-(4-nitrophenyl)−5-phenyltetrazolium chloride to form a red formazan dye. The SOD activity was measured by the degree of inhibition of this reaction.

### Cytological Method

The epididymides were separated from testes and adherent fatty tissues and ligaments were removed. Subsequently, the sperm suspension was isolated from the epididymides.

The sperm samples obtained from the epididymides were divided into two groups for examination. Sperm morphology was observed using Hancock solution [33, 34] with an Olympus CX41 Phase-Contrast microscope. Spermatozoa abnormalities, including head, neck, and tail abnormalities, were determined. According to this method, sperm with abnormalities were counted for at least 200 sperms in each sample, and the rates of sperm anomalies were expressed as percentages.

A portion of the obtained sperm samples was stained using eosin-nigrosin staining [35], and the examination of live-dead spermatozoa, and counting within 200 spermatozoa was performed using a Leica DMLB research microscope. Based on the staining in the head region, spermatozoa with a stained head were considered dead, while those without staining were considered alive.

### Statistical Analysis

The data were statistically analyzed using SPSS version 22 for Windows (serial number: 416a1604ed18748d3f27). Repeated measures analysis of variance (ANOVA) was used to analyze body weights. Paired *t*-tests were applied to compare body weight changes within groups over time. The chi-square test was applied to categorical data (caspase 3). For other data, the normality of the distribution was assessed using the Kolmogorov–Smirnov test. Differences between groups that did not exhibit a normal distribution were analyzed using the Kruskal–Wallis test. Post hoc pairwise multiple comparison tests were conducted to identify the source of significant differences. One-way ANOVA was used to evaluate differences between groups with a normal distribution, and the Duncan test was applied to determine the source of group differences [36]. Results with a significance level of *p* < 0.05 were considered statistically significant. All data are presented as mean ± standard error.

## Results

### Body and Testicular Weight

**Fig. 1:**
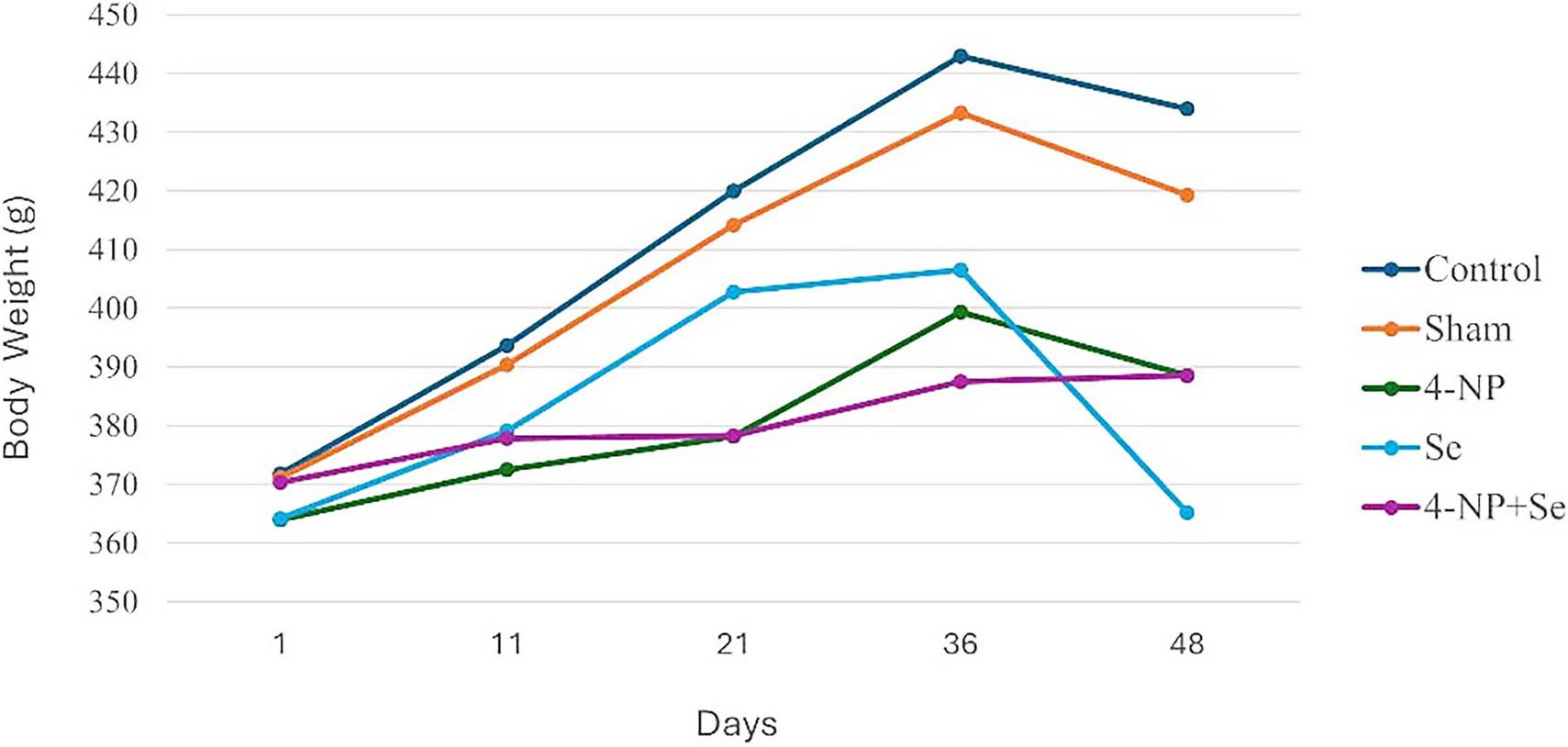
Changes in body weight (grams) across different time points for the control and experimental groups. Significant changes over time were observed in all groups (*P* < 0.001) as determined by repeated measures ANOVA. In the Se group, a significant decrease in body weight was observed between the 36th and 48th days (*P* < 0.05), as determined by a *t*-test

The body weights of rats were compared across days 1, 11, 21, 36, and 48 (Table 1). A repeated measures analysis of variance revealed a significant overall change in body weight over time (*P* < 0.001). Subsequent paired *t*-tests showed a significant decrease in body weight in the Se group between days 36 and 48 (*P* < 0.05) (Fig. 1).

**Table 1:** The control and experimental groups’ body weights (grams) were determined throughout the experiment

Day group (*n* = 6)	1st day (*x* ± *S x*)	11th day (*x* ± *S x*)	21st day (*x* ± *S x*)	36th day (*x* ± *S x*)	48th day (*x* ± *S x*)	*P* (change over time)
Control	371.83 ± 26.60	393.66 ± 25.17	420.00 ± 23.88	443.00 ± 26.70	434.00 ± 27.22	^***^
Sham	371.16 ± 19.06	390.33 ± 21.44	414.16 ± 21.60	433.33 ± 21.85	419.33 ± 22.53	^***^
4-NP	364.00 ± 14,06	372.50 ± 15.59	378.16 ± 12.08	399.33 ± 11.78	388.50 ± 13.05	^***^
Se^*^	364.16 ± 12.24	379.16 ± 11.98	402.83 ± 11.59	406.50 ± 9.75	365.16 ± 15.38	^***^
4-NP + Se	370.33 ± 12.17	377.83 ± 10.32	378.33 ± 8.80	387.50 ± 9.78	388.50 ± 13.05	^***^
*P*	NS	NS	NS	NS	NS	

*4-NP;* 4-nonylphenol, *Se;* selenium, *n;* number of rats, $\overline x$; mean, $S\overline x$; standard error of mean (SEM), *NS; *non-significant

^***^P < 0.001

^*^Se significant difference between the 36th and 48th days (*t*-test, *P* < 0.05)

Testicular weights (grams) and the testicular weight to body weight ratio (TW/BW × 100) for the control and experimental groups are shown in Table 2. No significant differences were observed in testicular weights between the groups (*P* > 0.05). However, the TW/BW ratio was significantly higher in the Se group compared to the other groups (*P* < 0.01), indicating a marked difference in body weight relative to testicular weight.

**Table 2:** Testicular weights (grams) and TW/BW (× 100) in control and experimental groups

Group (*n* = 6)	Right testes (*x* ± *S x*)	Left testes (*x* ± *S x*)	Total testes (*x* ± *S x*)	TW/BW (× 100) (*x* ± *S x*)
Control	1.70 ± 0.06	1.73 ± 0.06	3.43 ± 0.12	0.80 ± 0.03^b^
Sham	1.90 ± 0.06	1.91 ± 0.06	3.81 ± 0.12	0.92 ± 0.03^b^
4-NP	1.77 ± 0.07	1.78 ± 0.07	3.55 ± 0.14	0.92 ± 0.02^b^
Se	1.83 ± 0.08	1.83 ± 0.08	3.66 ± 0.16	1.01 ± 0.06^a^
4-NP + Se	1.73 ± 0.04	1.78 ± 0.04	3.51 ± 0.08	0.93 ± 0.02^b^
*P*	NS	NS	NS	^**^

*4-NP;* 4-nonylphenol, *Se;* selenium, *n;* number of rats, $\overline x$; mean, $S\overline x$; standard error of mean (SEM), *NS;* non-significant

^**^P < 0.01

### Histological Findings

**Fig. 2:**
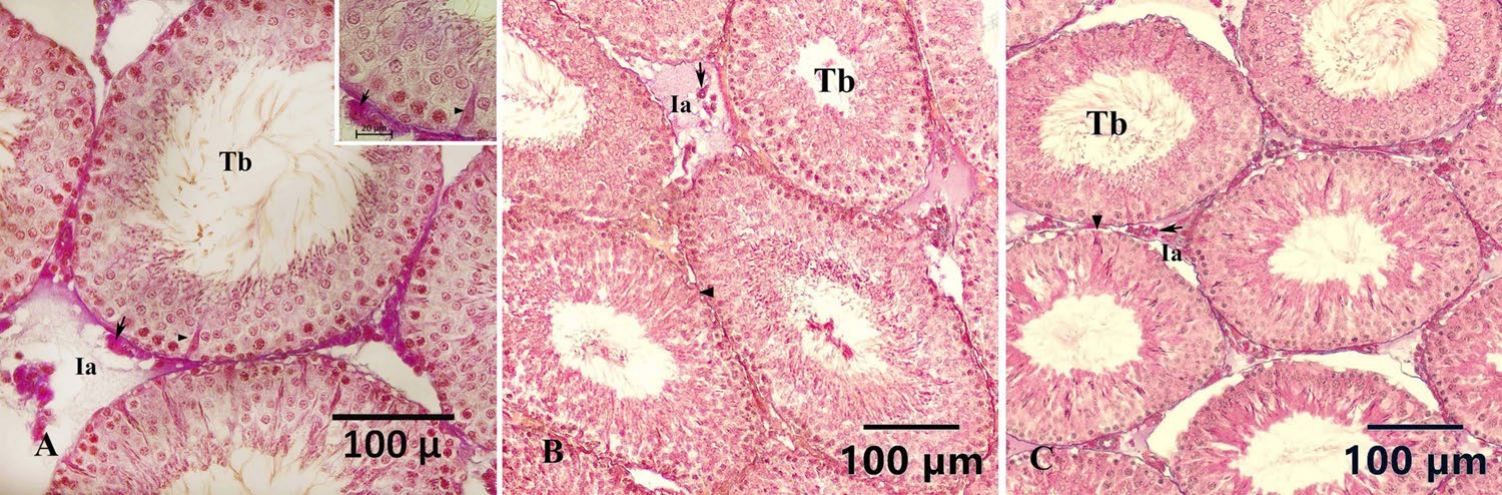
**A** Seminiferous tubules in the testis of the control group. **B** Appearance of seminiferous tubules in the testis of the Se group. **C** Seminiferous tubules in the testis of the 4-NP + Se group. Tb, seminifer tubule; Ia, intertubular area; arrowheads, Sertoli cells; arrow, Leydig cells. Triple staining method by Crossman

**Fig. 3:**
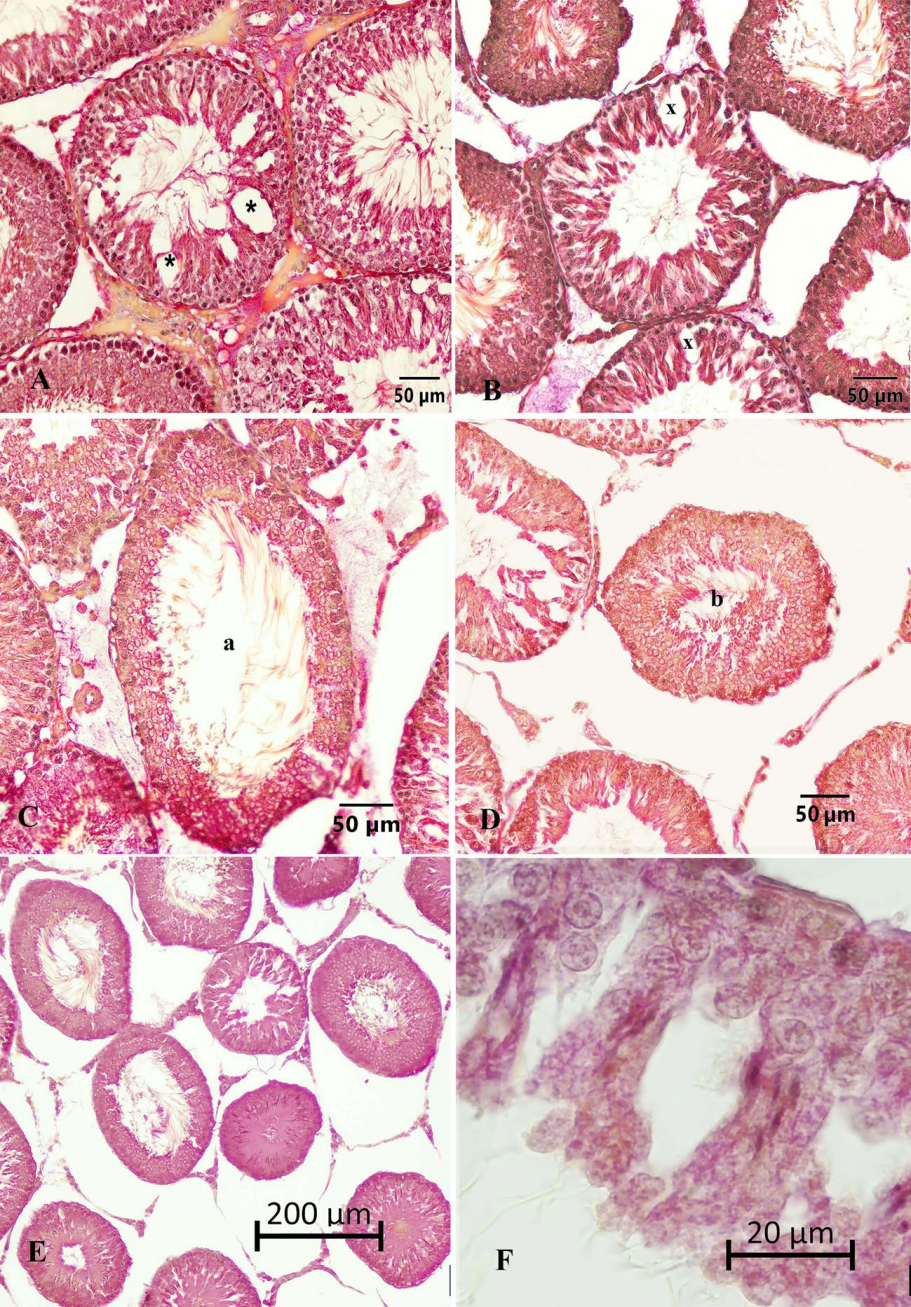
Histological changes observed in the testis of the 4-NP group: **A** Vacuole formation in the seminiferous tubules (*). **B** Shedding of germ cells ( ×). **C** Thinning of the spermatogenic epithelium (a). **D** Shrinking of the seminiferous tubules (b). **E** Edema in the interstitial space. **F** Close-up of epithelial shedding. Triple staining method by Crossman

**Fig. 4:**
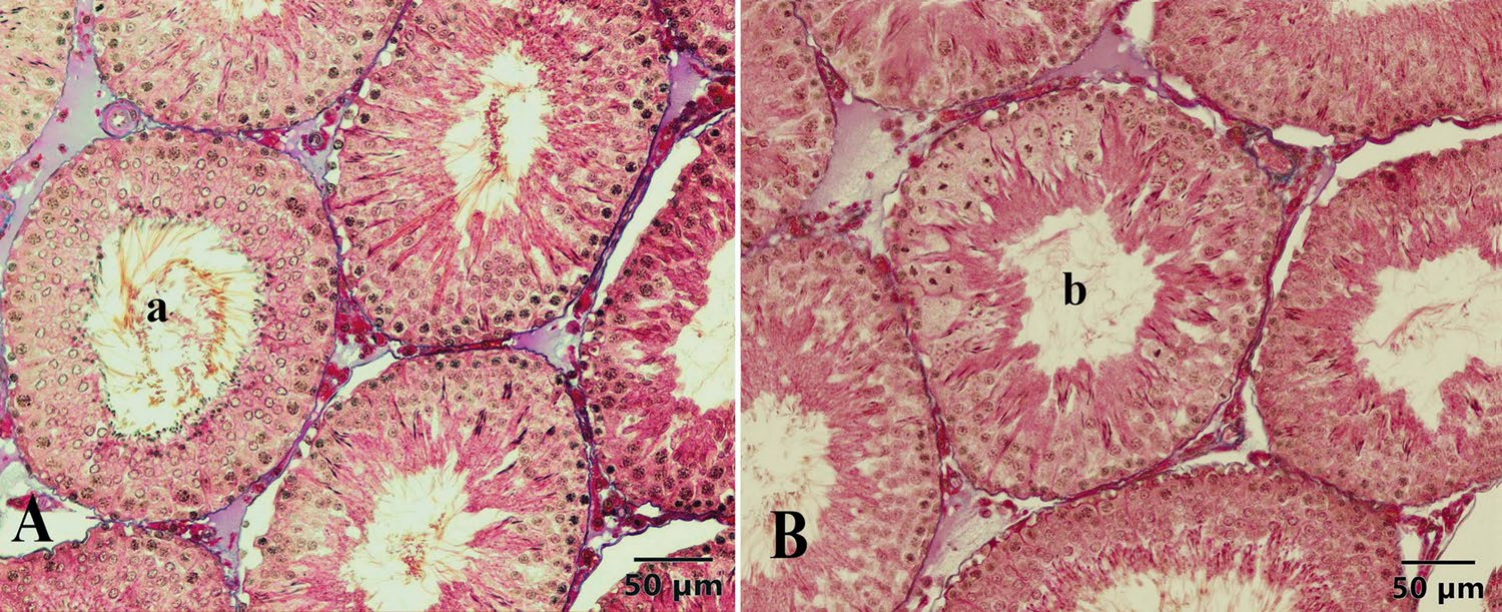
**A** A seminiferous tubule in the testis of the control group at the stages VII–VIII of the spermatogenic cycle (a). **B** A seminiferous tubule in the testis of the control group at stage XIV of the spermatogenic cycle (b). Triple staining method by Crossman

Images of seminiferous tubules in tissue sections from the control, Se, and 4-NP + Se groups are presented in Fig. 2. In sections of the control groups, the seminiferous tubules were well positioned, with Sertoli cells within the tubules and Leydig cells in the interstitial space distributed regularly, maintaining a normal overall appearance. The testicular tissue sections of the Se and 4-NF + Se groups generally resembled those of the control groups, although some histological changes such as epithelial shedding and vacuole formation were observed. Additionally, slight shrinkage of the tubules was noted in the 4-NF + Se group. In contrast, the rats in the 4-NP group exhibited more pronounced histological changes, including vacuole formation in the seminiferous tubules (Fig. 3A), shedded germ cells (Fig. 3B, F), thin spermatogenic epithelium (Fig. 3C), and reduced tubular diameter (Fig. 3D). Edema was observed in the interstitial space (Fig. 3E). Different stages of spermatogenesis could be distinguished in the tubular epithelium of the tissue sections (Fig. 4).

### Histomorphometric Findings

**Fig. 5:**
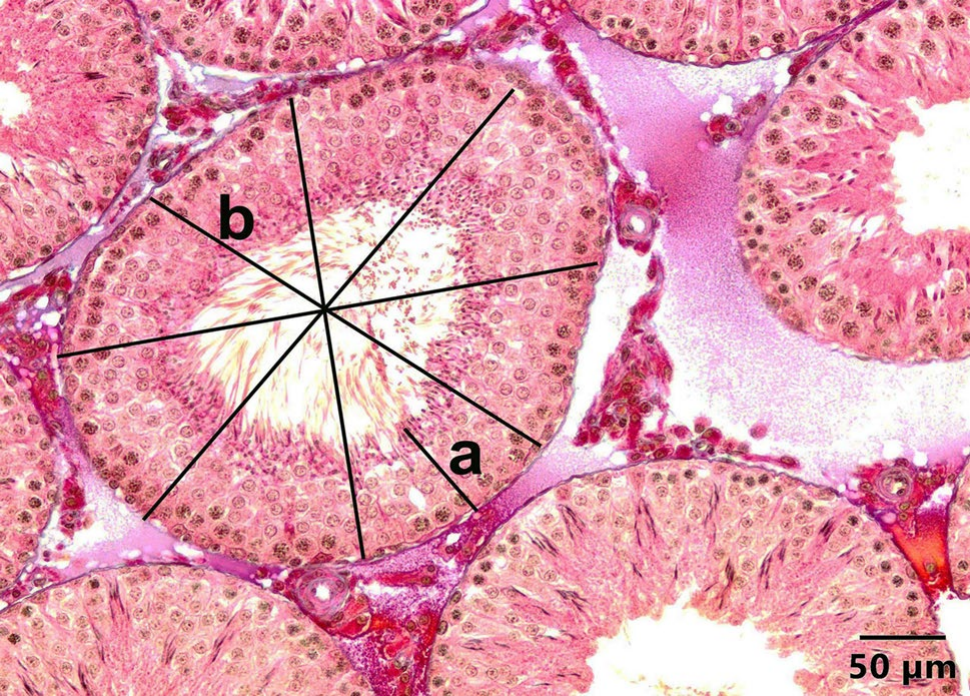
Measurement of epithelial height (**a**) and diameter (**b**) of a seminiferous tubule in the control group. Triple staining method by Crossman

Seminiferous tubule diameters and epithelial heights corresponding to stages VII–VIII were assessed in both control and experimental groups (Fig. 5). The obtained values are presented in Table 3. Examining the tubular diameter values revealed that the seminiferous tubule diameter in the 4-NP and Se groups was significantly lower compared to the control group, sham group, and the 4-NP + Se group, with the difference being statistically significant (*P* < 0.001). Notably, the tubular diameter in the 4-NP + Se group was similar to that of the control and sham groups.

**Table 3:** Seminiferous tubule diameter, epithelial height (µm), and density values of stage XIV tubules for the control, sham control, and experimental groups

Group (*n* = 6)	Tubule diameters (*x* ± *S x*)	Epithelial heights (*x* ± *S x*)	Density of stage XIV tubule (*x* ± *S x*)
Control	292.68 ± 5.09^a^	69.10 ± 1.44^a^	8.41 ± 0.38^c^
Sham	298.04 ± 5.98^a^	67.87 ± 1.61^a^	9.44 ± 0.26^b^
4-NP	270.20 ± 2.71^b^	52.38 ± 0.79^d^	7.66 ± 0.36^c^
Se	271.26 ± 3.69^b^	60.08 ± 0.92^c^	9.38 ± 0.32^b^
4-NP + Se	284.20 ± 3.63^a^	63.55 ± 0.85^b^	11.30 ± 0.33^a^
*P*	^***^	^***^	^***^

*4-NP;* 4-nonylphenol, *Se;* selenium, *n;* number of rats, $\overline x$; mean, $S\overline x$; standard error of mean (SEM)

^***^P < 0.001

^a,b,c,d^ Differences between group means represented by different letters in the same column are significant

Similarly, analysis of the tubule epithelial height (Table 3) showed that the value in the 4-NP group was significantly lower than in all other groups (*P* < 0.001). The epithelial height in the Se group was significantly lower than in the control, sham control, and 4-NP + Se groups but higher than in the 4-NP group (*P* < 0.001). Notably, the 4-NP + Se value was lower than in the control groups but higher than in the 4-NP and Se groups (*P* < 0.001).

The ratio (%) of stage XIV tubules was determined in the cross-sections and statistically evaluated (Table 3). Analysis showed that the stage XIV tubule density in the 4-NP group was significantly lower than in the sham control, Se, and 4-NP + Se groups (*P* < 0.001) but similar to the control group (*P* > 0.05). The highest value in terms of stage XIV tubule density was observed in the 4-NP + Se group, which was statistically significant (*P* < 0.001). The value for the Se group was higher than in the control group (*P* < 0.001) but similar to the sham group (*P* > 0.05). Additionally, the stage XIV tubule density in the sham control group was higher than in the control group (*P* < 0.001) (Table 3).

### Immunohistochemical Findings

#### Caspase 3

**Fig. 6:**
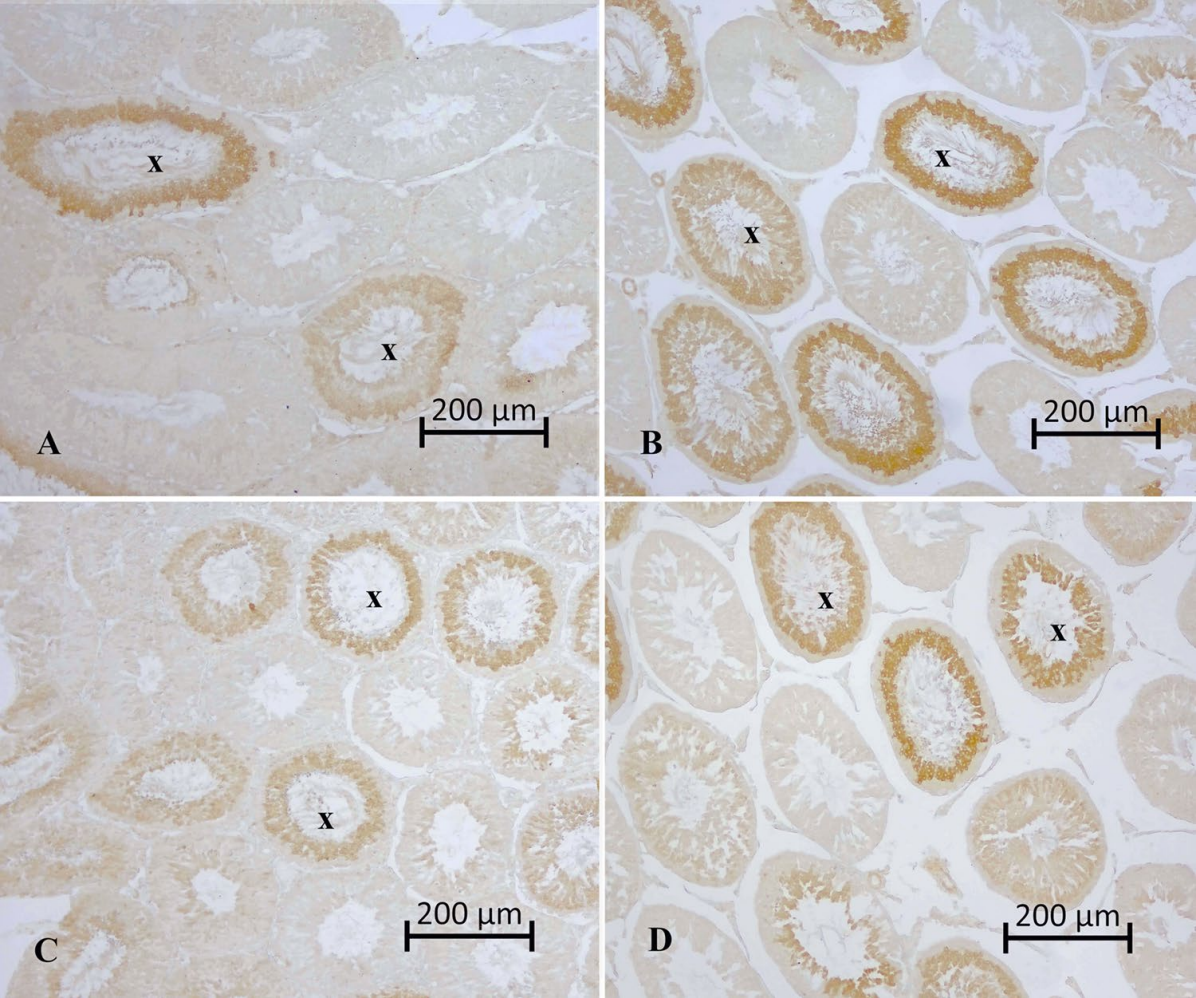
Caspase 3 positivity at the tubular level. **A** Caspase 3 intensity in the control group. **B** Caspase 3 intensity in the 4-NP group. **C** Caspas 3 intensity in the Se group. **D** Caspase 3 intensity in the 4-NP + Se group. x, tubules with marked caspase 3 staining. Strept ABC staining method

Caspase 3 density was examined in the sections. Intense caspase 3 positivity was observed in some tubules at the tubular level (Fig. 6). The percentages of tubules with significant caspase 3 density were determined in the sections (Table 4).

**Table 4:** Distribution (%) of tubules with intense caspase 3 positivity

Group (*n* = 6)	Distribution of intense caspase 3 positivity (%) (*x* ± *S x*)
Control	23.36 ± 0.60^d^
Sham	23.64 ± 0.56^d^
4-NP	49.72 ± 0.70^a^
Se	30.06 ± 0.55^c^
4-NP + Se	33.47 ± 0.56^b^
*P*	^***^

*4-NP;* 4-nonylphenol, *Se;* selenium, *n;* number of rats, $\overline x$; mean, $S\overline x$; standard error of mean (SEM)

^***^P < 0.001

^a,b,c,d^Differences between group means indicated by different letters in the same column are significant

The percentages of tubules with intense caspase 3 positivity were significantly lower in the control and sham control groups (*P* < 0.001). The 4-NP group exhibited the highest caspase 3 positive tubule density (*P* < 0.001). Furthermore, the density of intense caspase 3 positive tubules in the Se and 4-NP + Se groups was higher than that in the control groups (*P* < 0.001) (Table 4).

#### Connexin 43

**Fig. 7:**
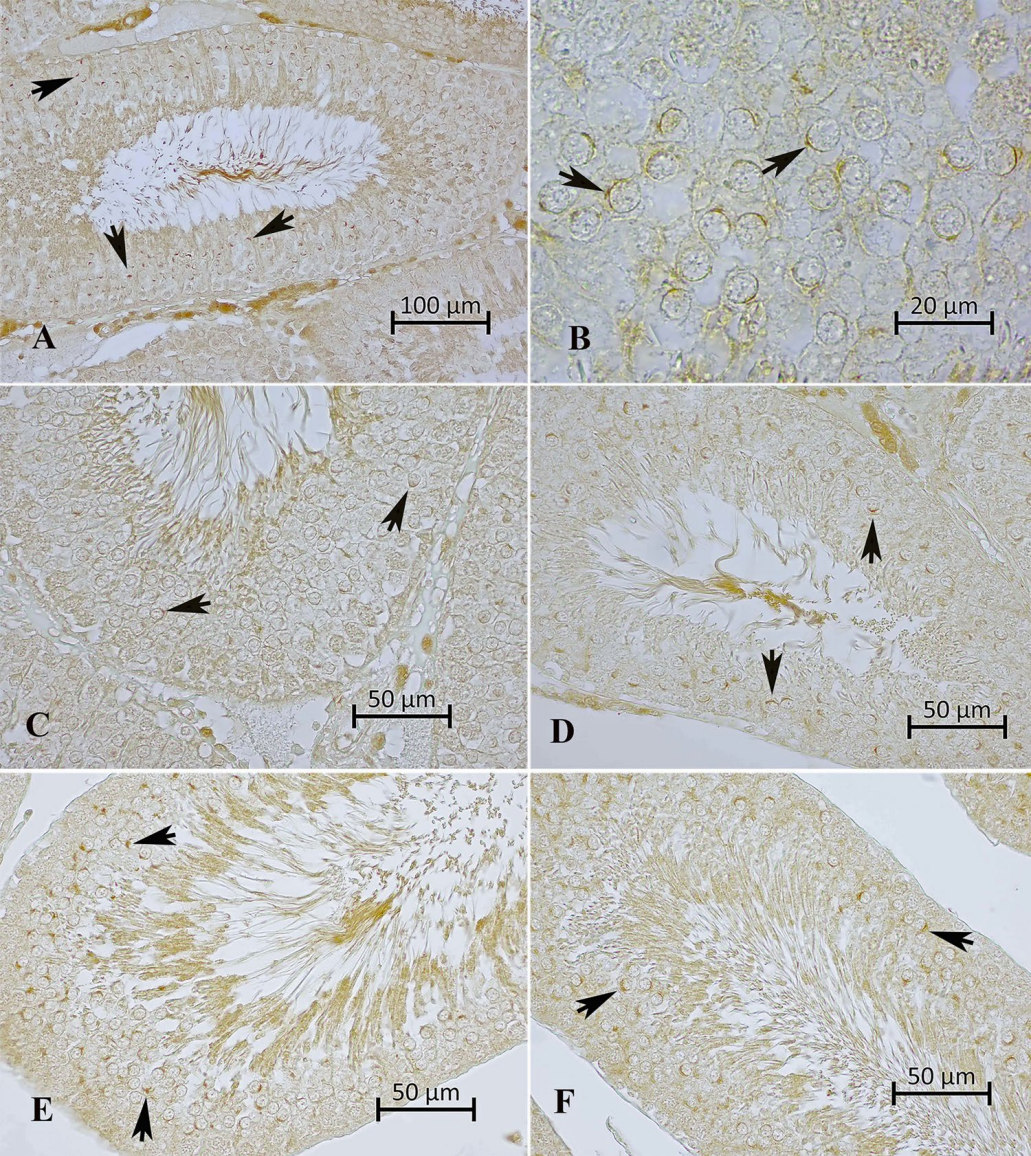
Appearance of CX 43 positivity in groups.** A** CX 43 density in the control group, **B** close-up of CX 43 positivity in the control group. **C** and **D** CX 43 density in the 4-NP group, **E** CX 43 density in the Se group. **F** CX 43 density in the 4-NP + Se group. Arrows, CX43 positivity. Strept ABC staining method

CX43 positivity is demonstrated in Fig. 7. In the control group, CX 43 positivity was observed to be higher compared to the 4-NP group. The CX43 positivity in the Se group was similar to that of the control group. In the 4-NP + Se group, although not as high as in the control group, CX 43 positivity was found to be more pronounced compared to the 4-NP group. It was noteworthy that CX43 positivity among Leydig cells was not affected by 4-NP.

### Biochemical Findings

MDA and SOD values were determined both in serum and testicular tissue (Table 5). The highest MDA value in serum was detected in the NP group (*P* < 0.05). According to the results, the serum MDA value of the 4-NP + Se group was not significantly different from the Se and control groups (*P* > 0.05). When examining the SOD value, the lowest value was determined in the 4-NP group (*P* < 0.01). While the highest SOD value was measured in the Se group (*P* < 0.01), no difference was observed between the values of the 4-NP + Se and control groups (*P* > 0.05) (Table 5).

**Table 5:** MDA and SOD levels in serum and testis

Groups (*n* = 6)	Serum MDA (nmol/mg protein) (*x* ± *S x*)	Serum SOD (U/ mg protein) (*x* ± *S x*)	Testis MDA (nmol/mg protein) (*x* ± *S x*)	Testis SOD (U/mg protein) (*x* ± *S x*)
Control	0.81 ± 0.08^b^	85.82 ± 1.94^b^	222.51 ± 8.97^b^	5.33 ± 0.57^b^
Sham	0.80 ± 0.04^b^	85.43 ± 4.12^b^	210.22 ± 15.80^b^	5.12 ± 0.53^b^
Se	0.75 ± 0.05^b^	101.62 ± 5.94^a^	176.30 ± 5.03^c^	6.26 ± 0.17^a^
4-NP	1.10 ± 0.05^a^	53.25 ± 7.39^c^	303.72 ± 22.29^a^	2.71 ± 0.45^c^
4-NP + Se	0.81 ± 0.07^b^	78.34 ± 5.77^b^	202.81 ± 16.03^bc^	5.79 ± 0.32^ab^
*P*	^*^	^**^	^**^	^**^

*4-NP;* 4-nonylphenol, *Se;* selenium, *n;* number of rats, $\overline x$; mean, $S\overline x$; standard error of mean (SEM)

^*^P < 0.05

^**^P < 0.01

^a,b,c^Different letters within the same column indicate statistically significant differences

In testicular tissue (Table 6), the highest MDA level (*P* < 0.001) and the lowest SOD amount (*P* < 0.001) were observed in the 4-NP group. Conversely, the lowest MDA value was found in the Se and 4-NP + Se groups (*P* < 0.001). The MDA data of the control groups and the 4-NP + Se group exhibited similarities. The highest SOD value was noted in the Se and 4-NP + Se groups (*P* < 0.001). Notably, SOD data of the control groups and the 4-NP + Se group were similar (Table 5).

**Table 6:** Abnormal sperm counts and sperm viability

Groups**(***n*** = **6)	Abnormal spermatozoa (%)**(***x* ± *S x***)**	Sperm viability (%**) (***x* ± *S x***)**
Control	34.00 ± 3.91^b^	82.73 ± 1.29^a^
Sham	34.87 ± 1.63^b^	75.87 ± 3.67^ab^
4-NP	75.93 ± 3.72^a^	34.20 ± 2.43^c^
Se	32.60 ± 1.13^b^	62.87 ± 7.73^b^
4-NP + Se	43.20 ± 6.05^b^	69.07 ± 3.20^b^
*P*	^***^	^***^

*4-NP* 4-nonylphenol, *Se* selenium, *n* number of rats, $\overline x$; mean, $S\overline x$; standard error of mean (SEM)

^***^P < 0.001

^a,b^Differences between group means indicated by different letters in the same column are statistically significant

### Sperm Morphology

#### Normal/Abnormal Spermatozoa Evaluation

Abnormal spermatozoa were classified as head, midpiece, “S”-shaped, and tail abnormalities. The results of normal-abnormal spermatozoa count for each group were determined (Table 6).

In the sperm samples of the 4-NP group, a significant anomaly was observed, primarily in the form of tail abnormalities. When compared to the other groups, the highest anomaly rate was seen in the 4-NP group (*P* < 0.001). It was found that the number of abnormal spermatozoa in the Se and even the 4-NP + Se groups was similar to that of the control groups (*P* > 0.05) (Table 6).

#### Sperm Viability Evaluation

The stained spermatozoa in the prepared smears were categorized as dead if they were stained and live if they were unstained. The numbers of dead and live spermatozoa for each group were determined. When the evaluation of dead-live spermatozoa was performed on 200 spermatozoa in the prepared smears, it was observed that the number of live spermatozoa was the lowest in the 4-NP group (*P* < 0.001) (Table 6). The values for the control groups were similar to each other, and it was noted that the live spermatozoa count in the Se and 4-NP + Se groups was also similar to that of the sham control group (*P* > 0.05) (Table 6).

## Discussion

In this study, we investigated whether selenium, known for its antioxidant properties, protects against or mitigates oxidative damage in testicular toxicity induced by 4-NP in rats.

### Effects of 4-NP and Se on Body and Testis Weights

Body weight showed significant changes over time (*P* < 0.001) across all groups. This could be attributed to various factors such as age, growth, or effects of treatment. Paired *t*-tests indicated a significant weight decrease in the Se group between days 36 and 48 (*P* < 0.05).

Raines and Sunde [37] reported that rats fed a diet containing 5 µg Se/g exhibited lower body weight starting from the 10th day. Jia et al. [38] reported that the level of selenite and high Se protein with no observed adverse effects in Sprague–Dawley male and female rats was 2 ppm Se, corresponding to 0.14 mg/kg body weight/day for males and 0.20 mg/kg body weight/day for females. They observed a reduction in body weight when this amount was exceeded. In our study, selenium was administered at a dose of 0.5 mg/kg/day, by the literature [26]. In contrast to these findings, Antunes et al. [39] found no weight difference when 1 mg/kg selenium was administered.

The significantly higher TW/BW ratio in the Se group compared to the other groups (*P* < 0.01) is particularly interesting (Table 2), especially considering the body weight loss observed in this group (Fig. 1). The increased TW/BW ratio, despite body weight loss, may indicate testicular mass preservation, suggesting selenium’s protective role against 4-NP toxicity.

Han et al. [25] observed that administering 125 and 250 mg/kg 4-NP for 6 weeks reduced testis and epididymis weights in 4-week-old rats, with greater reductions at higher doses. Similarly, Chitra et al. [40] found that daily doses of 1, 10, and 100 µg/kg of 4-NP over 45 days caused similar reductions in testicular weights in albino male rats. Sakr et al. [41] reported that exposure to carbimazole at 1.35 mg/kg for 8 weeks led to weight decreases, but selenium treatment afterward resulted in increased testis and body weights. Variations in results may be attributed to differences in rat age, administration duration, or 4-NP solvent.

### Effects of 4-NP and Se Treatment on Testicular Histology

Histological analysis in the 4-NP group revealed significant structural disruptions, aligning with existing literature that associates environmental toxicants with testicular damage. Notably, changes such as vacuolization, germ cell loss, and reduced tubular diameters reflect compromised spermatogenesis and impaired testicular function. Lu et al. [42] and Kim et al. [43] also found dose-dependent testicular degeneration and a reduction in seminiferous tubule volume, diameter, and germ cells in rats and mice, respectively, supporting the negative effects of 4-NP exposure on testicular health.

In this study, the Se and 4-NP + Se groups generally showed similar testicular tissue characteristics to controls, with slight histological changes, such as epithelial shedding and vacuole formation. However, co-administration of selenium with 4-NP improved tubule diameter and epithelial height, resembling control groups. This suggests selenium may protect against NP toxicity, potentially through its antioxidant properties, as shown in studies by Antunes et al. [39] and Sakr et al. [41]. Another study [44] found selenium improved seminiferous tubule diameter in a testicular torsion model. The reduced tubule diameter and epithelial height in the Se group compared to controls may result from variations in selenium dose and duration.

When evaluating the stage XIV percentage (%) tubule density values, it was found that the sham control group exhibited a higher value compared to the control group (*P* < 0.001). This increase may be linked to the corn oil administered to the sham control group, which is commonly used as a vehicle for delivering lipophilic chemicals in toxicity studies. Refined corn oil is composed of 99% triacylglycerols, including polyunsaturated, monounsaturated, and saturated fatty acids [45].

Naji [46] found that male rabbits given 2–2.5 ml of corn oil daily for 50 days showed significant improvements in sperm parameters, including testicular sperm concentration and motility (*P* < 0.05). While the dose administered by Naji [46] was higher, the sham control group in the present study received 0.5 ml of corn oil for 48 days. The major components of corn oil include polyunsaturated fatty acids such as linoleic acid, monounsaturated oleic acid, and a small amount of saturated fatty acids, with a total triglyceride content of about 95%. Given its widespread use in toxicity and dietary studies, corn oil may have a beneficial effect on reproductive health. Yan et al. [47] also reported that an appropriate n-3/n-6 PUFA ratio in rat diets improved reproductive performance and strengthened testicular and sperm structural integrity.

In the Se-treated group, stage XIV tubule density was similar to the sham control and significantly higher than the control group (*P* < 0.001), indicating selenium enhances tubule density during this stage. Selenium plays a crucial role in biochemical and physiological processes, especially in antioxidant defense [48]. It is vital for normal spermatogenesis in mammals, with effects linked to PHGPx/GPx4 and selenoprotein P [18]. Taghizadeh et al. [49] observed no significant changes in rat testes with 0.4 mg/kg sodium selenite daily for 60 days. In contrast, our study found that selenium supplementation significantly increased stage XIV tubule density (*P* < 0.001). The use of corn oil in the Se group as a solvent vehicle may have enhanced selenium’s effects. The highest stage XIV tubule density in the 4-NP + Se group can be attributed to both the selenium effect and the use of corn oil as the solvent. Although the 4-NP group had the lowest stage XIV tubule density, the significant difference from the control group might be due to the beneficial effects of unsaturated fatty acids in the corn oil, as observed in the sham control group [46].

### Effects of 4-NP and Se on Oxidative Stress Parameters

Our study found the highest MDA levels (serum: *P* < 0.05, tissue: *P* < 0.001) and the lowest SOD levels (serum: *P* < 0.01, tissue: *P* < 0.001) in the 4-NP group, indicating that 4-NP induces oxidative stress and reduces antioxidant levels. Oxidative stress disrupts spermatogenesis and sperm function, potentially causing infertility [50]. Excessive ROS increases mitochondrial damage and activates apoptotic pathways [51]. Studies [40, 52] have shown that NP disrupts cytochrome P450, resulting in ROS buildup, lipid peroxidation, and reduced antioxidants. Shalaby and Saleh [53] found that 100 mg/kg NP decreased plasma antioxidants and increased testicular MDA in rats. Our MDA and SOD data in serum and testicular tissue are consistent with these findings.

On the other hand, MDA and SOD levels from both serum samples and testicular tissue in the 4-NP + Se group were similar to the control groups (*P* > 0.05), indicating selenium’s protective role against 4-NP toxicity and its support for antioxidants. Previous studies [54, 55] highlight selenium’s key role in testosterone biosynthesis, sperm formation, and H2O2 and lipid hydroperoxide metabolism. Selenium is crucial for enzymes like GPx, deiodinase, and TRxR, which defend cells from oxidative damage [48]. Taghizadeh et al. [49] showed that sodium selenite treatment restored testicular damage in varicocele rats, while no changes were observed in normal rats.

### Effects of 4-NP and Se on Caspase-3 Positivity in Testicular Tissue

Caspase-3, a key apoptosis marker, is activated in cells through both extrinsic (death ligands) and intrinsic (mitochondrial) pathways [56]. In this study, the highest caspase-3 positive tubule density was observed in the 4-NP group (*P* < 0.001), with the lowest in the control and sham control groups (*P* < 0.001), indicating that NP negatively affects spermatogenesis. Similar studies have shown that NP increases apoptosis markers such as Fas, FasL, TUNEL-positive cells, and caspase mRNA expression and induces ER stress in testicular cells [57–59].

Caspase-3 positive tubule density in the Se and 4-NP + Se groups was higher than in the controls (*P* < 0.001). Ranawat and Bansal [60] showed that selenium imbalance activates caspase-3 and apoptosis via ROS production. The higher caspase-3 density in selenium-treated groups suggests potential selenium toxicity. However, MDA data from serum and testis do not indicate toxicity, consistent with Taghizadeh et al. [49], who observed no significant changes in antioxidant activity or MDA levels after 0.4 mg/kg sodium selenite treatment for 60 days. In contrast, selenium administration at 0.5 mg/kg/day significantly increased SOD levels in both serum (*P* < 0.01) and testicular tissue (*P* < 0.001) compared to the control groups, while MDA levels were significantly lower than in the 4-NP group, indicating selenium’s protective effect against oxidative damage.

The observation of caspase-3 positivity at the tubular level in the Se group despite low MDA levels suggests that caspase-3 expression may play a role beyond apoptosis in regulating the growth and homeostatic maintenance of both normal and malignant cells, as indicated by recent studies [56, 61].

### Effects of 4-NP and Se on CX43 Expression in Testicular Tissue

CX43 localization was evaluated subjectively using photographs of the areas with the highest intensity of staining, as the positive regions were too small for reliable counting or scoring. In future studies, biochemical methods could provide a more objective evaluation. CX43 positivity was reduced in the 4-NP-treated groups, while the 4-NP + Se group showed some protective effect of selenium. Aravindakshan et al. [62] reported that NP exposure in rat testes (1–50 µM for 24 h) disrupted intercellular communication, reducing CX43 levels.

CX43 expression in Leydig cells was unaffected by 4-NP. Interestingly, Chojnacka et al. [63] found that flutamide exposure increased CX43 expression in hypertrophic Leydig cells, linked to cell growth. They noted that flutamide affected Leydig cells differently from other testicular cells.

### Effects of 4-NP and Se on Sperm Abnormalities and Viability

Selenium’s effects on sperm viability and morphology were examined due to its antioxidant properties against 4-NP toxicity. The 4-NP group had the lowest viable sperm count (*P* < 0.001) and a significantly higher proportion of abnormal sperm (*P* < 0.001). In contrast, sperm viability and abnormalities in the Se and 4-NP + Se groups were similar to the control group (*P* > 0.05), suggesting selenium mitigates 4-NP-induced damage. Han et al. [25] reported that oral administration of 125 and 250 mg/kg NP for 50 days in male rats reduced epididymal sperm density, while Seema et al. [64] found that selenium improved sperm count, motility, and restored testicular enzyme activities in rats treated with nicotine.

In terms of live spermatozoon count, the control and sham control groups showed similar values (*P* > 0.05). However, counts in the Se and 4-NP + Se groups were lower than controls (*P* < 0.001) but similar to sham controls (*P* > 0.05), suggesting selenium mitigates 4-NP toxicity. The lower count in the Se group may relate to dosage or duration. Corn oil in the sham group may also contribute to sperm count [46].

## Conclusion

In conclusion, this study identified testicular toxicity associated with the widely encountered 4-NP through cytological, histological, histometric, immunohistochemical, and biochemical assessments. Selenium was found to have a protective effect against the induced toxicity, as evidenced by the increased stage XIV tubule density in the 4-NP + Se group, higher caspase-3 levels in the 4-NP group compared to the 4-NP + Se group, reduced MDA levels, and increased SOD levels in both serum and testis tissue, as well as improved sperm viability and increased normal spermatozoa count. Additionally, the observed decrease in weight gain, reduction in tubule diameter and epithelial height, and higher caspase-3 levels in the Se group compared to the control groups suggest that selenium may also have metabolic adverse effects related to dosage or duration of use, which should be carefully considered. Future research should continue to explore the effects of selenium and determine the necessary parameters to minimize its adverse effects.

## Data Availability

No datasets were generated or analysed during the current study.
